# Decreased photosynthetic rate under high temperature in wheat is due to lipid desaturation, oxidation, acylation, and damage of organelles

**DOI:** 10.1186/s12870-018-1263-z

**Published:** 2018-04-05

**Authors:** M. Djanaguiraman, D. L. Boyle, R. Welti, S. V. K. Jagadish, P. V. V. Prasad

**Affiliations:** 1 Department of Agronomy, 2004 Throckmorton Plant Sciences Center, Kansas State University, Manhattan, KS 66506 USA; 2 Division of Biology, Kansas State University, Manhattan, KS 66506 USA; 3 Department of Crop Physiology, Tamil Nadu Agricultural University, Coimbatore, India

**Keywords:** Anatomy, High temperature, Lipids, Oxidative damage, Photosynthesis, Wheat

## Abstract

**Background:**

High temperature is a major abiotic stress that limits wheat (*Triticum aestivum* L.) productivity. Variation in levels of a wide range of lipids, including stress-related molecular species, oxidative damage, cellular organization and ultrastructural changes were analyzed to provide an integrated view of the factors that underlie decreased photosynthetic rate under high temperature stress. Wheat plants of cultivar Chinese Spring were grown at optimum temperatures (25/15 °C, maximum/minimum) until the onset of the booting stage. Thereafter, plants were exposed to high temperature (35/25 °C) for 16 d.

**Results:**

Compared with optimum temperature, a lower photosynthetic rate was observed at high temperature which is an interplay between thylakoid membrane damage, thylakoid membrane lipid composition, oxidative damage of cell organelle, and stomatal and non-stomatal limitations. Triacylglycerol levels were higher under high temperature stress. Polar lipid fatty acyl unsaturation was lower at high temperature, while triacylglycerol unsaturation was the same at high temperature and optimum temperature. The changes in lipid species indicates increases in activities of desaturating, oxidizing, glycosylating and acylating enzymes under high temperature stress. Cumulative effect of high temperature stress led to generation of reactive oxygen species, cell organelle and membrane damage, and reduced antioxidant enzyme activity, and imbalance between reactive oxygen species and antioxidant defense system.

**Conclusions:**

Taken together with recent findings demonstrating that reactive oxygen species are formed from and are removed by thylakoid lipids, the data suggest that reactive oxygen species production, reactive oxygen species removal, and changes in lipid metabolism contribute to decreased photosynthetic rate under high temperature stress.

**Electronic supplementary material:**

The online version of this article (10.1186/s12870-018-1263-z) contains supplementary material, which is available to authorized users.

## Background

Wheat (*Triticum aestivum* L*.*) is grown in about 30% of the world’s area cultivated with cereals, occupying over 220 million hectares worldwide of which 50% of the area experiences high temperature (HT) stress [1]. In fact, global mean surface air temperature has increased by 0.8 °C in the twentieth century and is predicted to increase further by 3–5 °C by the end of twenty-first century [2]. An increase in number of hot days and temperature variability is also predicted. Wheat is very sensitive to HT during its floral development and grain-filling phase [3, 4]. The optimal temperature (OT) for anthesis and grain filling ranges from 12 to 22 °C for wheat [5], and grain yield is significantly reduced with HT [6]. Keeping in view the predicted increase in growing season temperature in wheat producing areas, it is important to understand mechanisms of HT tolerance in wheat to maintain and improve yield potential.

HT stress causes damage to thylakoid membranes [7] and decreases photosystem II (PSII) quantum yield [8, 9] and photosynthesis [10]. Apart from this, HT increases production of reactive oxygen species (ROS) including superoxide radical (O_2_^−^) and hydrogen peroxide (H_2_O_2_) and increase lipid peroxidation and cause membrane damage [11]. Elevated temperatures decrease the activities of antioxidant enzymes like superoxide dismutase (SOD), catalase (CAT) and peroxidase (POX) [11–13]. The impacts of HT stress on leaf level photosynthesis is known, however, the mechanisms driving tolerance or susceptibility based on various membrane lipid species (polar lipids, triacylglycerol, oxidized lipids and acylated galactolipids), ultrastructure of cell organelles and its association with oxidants and antioxidant enzyme activity are not well understood.

Membranes are the targets of HT stress and membrane lipid composition is a crucial factor for temperature tolerance or susceptibility. The membrane plays important roles in sensing environmental change, signal transduction and substance metabolism. The thylakoid membranes are the location for the light-dependent reactions for photosynthesis. In wheat, galactolipids (monogalactosyldiacylglycerol; MGDG and digalactosyldiacylglycerol; DGDG) are the major chloroplast lipids, and trienoic species of MGDG and DGDG are highly vulnerable to peroxidation by ROS and by lipoxygenase [14, 15]. The fate of these lipid species under HT stress is not fully understood. Additionally, membranes may serve both as sources of ROS during plant stress and as reservoirs to take up ROS [15–18]. In Arabidopsis, oxidized lipids are produced enzymatically through the action of lipoxygenase as well as non-enzymatically through the action of ROS [19]. Similar to ROS, oxidized lipids may act as signaling molecules that initiate stress responses in plants [20–24]. Earlier studies have shown that triacylglycerols (TAGs) are accumulated under HT stress [25]; however, the changes in the saturation index of these TAG under HT stress are not known.

Several studies indicate that HT stress increases membrane damage and decreases antioxidant levels in wheat at the seedling stage [26–29], anthesis stage [30], or grain filing stage [31]. Temperature-induced changes in membrane fluidity is one of the immediate consequences of temperature changes, including HT stress, in plants [32, 33]. The significance of membrane fluidity in temperature tolerance has been elucidated by mutation analysis and transgenic and physiological studies. In soybean, a mutant deficient in fatty acid unsaturation showed strong tolerance to HT [34]. Similarly, the thylakoid membranes of an Arabidopsis mutants deficient in ω-6 fatty acid unsaturation (*fad6*) showed increased stability at HT [35], and decreased lipid unsaturation in tobacco caused by silencing an ω-3 desaturase gene rendered the plants more tolerant to HT [36].

Although leaf ultrastructural changes may reflect the effects of HT stress, there is scant description of the anatomical and ultrastructural changes in wheat leaves under HT stress. High temperature stress increased the thicknesses of the palisade and spongy layers, and disrupted the plasma, chloroplast, and thylakoid membranes in soybean leaves leading to lack to integrity [37]. Disorganized thylakoids with a reduced thickness of grana stacking, decreased size of starch granule and increased numbers of plastoglobules [38], empty mitochondria and damaged PSII [39] have been observed under HT stress in grape leaves. Corresponding observations of wheat leaf anatomy have not been well explored and less understood.

HT during the wheat reproductive stage causes decreased seed set, grain number, grain filling duration, grain filling rate and individual grain weight [40–43]. Together, the effects of HT stress result in decreased grain yield and harvest index [5, 41, 42, 44, 45]. In this study, various physiological and anatomical parameters that can change under HT stress, namely oxidants, antioxidant enzyme activities, cell organelle structure, and corresponding lipid changes, are quantified to determine the mechanisms that decrease photosynthetic rate under HT stress. The study fills a knowledge gap by ascertaining the series of physiological changes, by recording wheat leaf ultrastructural changes caused by HT, and by linking these with biochemical changes related to polar, oxidized, and neutral lipids.

## Methods

Two independent experiments using Chinese Spring were conducted in controlled-environment facilities at Kansas State University, Department of Agronomy, Manhattan, KS, USA.

### Plant husbandry and growth conditions

Seeds of Chinese Spring wheat were sown at 4-cm depth in 1.8 L pots (pot diameter at the top and the bottom was 21 and 16 cm, respectively; pot depth was 20 cm) containing commercial Sun Grow Metro Mix 200 potting soil (Hummert International, Topeka, KS, USA). After emergence, plants were thinned to two plants per pot and maintained until maturity. A systemic insecticide, Marathon 1% G (granules) (i.e., Imidacloprid,1-[(6-chloro-3-pyridinyl)methyl]-N-nitro-2-imidazolidinimine, Hummert International, Topeka, KS, USA), was applied to each pot at 4 g per pot. The medium was fertilized with Osmocote at 5 g pot^− 1^ (controlled release plant food, 14:14:14% N: P_2_O_5_: K_2_O, respectively; Hummert International, Topeka, KS, USA) before sowing. Plants were watered daily to avoid water stress. The seedlings were grown in growth chambers (Conviron Model E15, Winnipeg, MB, Canada) maintained at optimum temperature (OT; 25/15 °C daytime maximum/nighttime minimum temperature), 14-h photoperiod, and 80–85% relative humidity. Each growth chamber was 136 cm wide, 246 cm long, and 180 cm high. The temperature regimes, i.e., the daytime maximum and nighttime minimum temperatures, were each held for 8 h; the transition periods between maximum and minimum temperatures were each 4 h. In all growth chambers, the canopy-level, photosynthetically-active radiation was about ~ 900 μmol m^− 2^ s^− 1^ provided by cool white fluorescent lamps (Philips Lighting Co., Somerset, NJ, USA). Plants in each growth chamber were randomly moved once in every 7 d to avoid positional effects within the chamber. To avoid water stress, all pots were kept in trays containing about 2-cm deep water from sowing to maturity.

At the completion of booting stage (Feekes growth stage 10.0), the plants were transferred to OT (25/15 °C, daytime maximum /nighttime minimum temperature, 25 °C: OT for spikelet initiation and anthesis [46]) or HT (35/25 °C daytime maximum /nighttime minimum temperature, > 34 °C: critical maximum temperature [47]. Apart from this, the OT thresholds for spring wheat was estimated to be 18 and 34 °C; above which an additional degree day is associated with a ~ 7% yield reduction]. The temperature treatments were randomly assigned to two growth chambers. Each growth chamber had 30 pots (60 plants). Out of these 60 plants, 40 plants were used for the measurement of physiological and biochemical traits at regular intervals (3 d), and ten plants were used to collect samples for lipid extraction and anatomical studies. The remaining ten plants were used for analyzing yield and yield components. For each physiological and biochemical measurement, five plants were individual replicates and the final number of samples was ten (5 plants × 2 experiments; *n* = 10). For lipid and anatomy studies, there were ten replications (5 plants × 2 experiments; *n* = 10). Yield and its components were the mean of 20 observations (10 plants × 2 experiments; *n* = 20). During the stress period, the position of pots was changed randomly every day in all growth chambers to avoid positional effects. The plants were maintained in the HT regime for 16 d. After that, they were returned to the original growth chamber (OT: 25/15 °C), where they remained till maturity. In both temperature regimes, day-maximum and night-minimum temperatures were held for 8 h, and the transition period between maximum and minimum temperatures was 4 h. Relative humidity in all growth chambers was set and maintained at 70–80%. In the future, it is predicted that increased temperature will be observed; however, the relative humidity will remain constant [48]. Air temperature and relative humidity were continuously monitored at 15-min intervals in all growth chambers throughout the experiment using a HOBO data logger (Onset Computer Corporation, Bourne, MA, USA). The photoperiod was 14 h, and photon flux density (400 to 700 nm) provided by cool fluorescent lamps was about 900 μmol m^− 2^ s^− 1^ at the top of the plant canopy. After the completion of Experiment I, the same two growth chambers were used for Experiment II with the same temperature, relative humidity, and light settings. The crop husbandry, temperature regimes, and traits recorded were the same as described below.

### Chlorophyll index, chlorophyll *a* fluorescence, and gas exchange measurements

At the completion of the heading stage, the main stem of each of 40 plants in each growth chamber was tagged for the measuring of physiological and biochemical traits. All physiological traits were measured on attached fully expanded flag leaves. In both experiments, chlorophyll index, chlorophyll *a* fluorescence, and gas exchange measurements were measured from five tagged flag leaves at OT and HT on days 0, 3, 6, 9 and 12 after the start of temperature treatments between 10:00 and 14:00 h. Chlorophyll index was measured using a self-calibrating chlorophyll meter (Soil Plant Analytical Device [SPAD], Model 502, Spectrum Technologies, Plainfield, IL). Thylakoid membrane stability was assessed by measuring chlorophyll *a* fluorescence using a fluorometer (OS5p, OptiScience, Hudson, NH) after 30 min of dark adaptation of leaves and by determining the ratio of basal fluorescence to maximum fluorescence. Increase in this ratio indicates damage to thylakoid membranes [49]. For other chlorophyll *a* fluorescence measurements, the leaves were dark adapted for 24 h to attain a maximum level of maximum fluorescence and a minimum level of heat dissipation [50]. The leaves were continuously irradiated with white actinic light to measure the initial fluorescence in leaves acclimated to irradiation (Fo′), steady-state fluorescence yield (Fs), and maximum fluorescence yield (Fms) of irradiated leaves. By using the above parameters the following chlorophyll *a* fluorescence parameters were calculated: effective quantum yield of PSII (ϕ PSII = [Fms-Fs]/fms); apparent rate of photochemical transport of electrons through PSII (ETR = ϕ PSII × PAR × 0.5 × 0.84), the coefficient of photochemical quenching (qP = [Fms-Fs]/[Fms -Fo]), and the coefficient of non–photochemical quenching of excitation energy (NPQ = [Fm- Fms]/fms) were calculated by the instrument software [50, 51]. In addition, leaf level gas exchange measurements (photosynthesis and stomatal conductance) were measured in five leaves using a LICOR 6400 portable photosynthesis system (LICOR, Lincoln, NE). Gas exchange measurements were taken at daytime growth temperature and ambient CO_2_ conditions (400 μmol mol^− 1^). Constant temperature within the chamber was maintained, using the built-in software of the instrument. The internal light-emitting diode (LED) light source in the LICOR 6400 was set at 1600 μmol m^− 2^ s^− 1^ to ensure a constant, uniform light across all measurements.

### Leaf collection for xanthine oxidase enzyme activity, hydrogen peroxide radical content, malondialdehyde content, and cell membrane stability

After recording the above physiological traits at day 0, 3, 6, 9 and 12 of the temperature treatment, the first, second and third leaves from the top were excised and immediately frozen in liquid nitrogen and stored in − 80 ^ο^C until further biochemical analyses, which are described in the following sections.

### Xanthine oxidase enzyme activity

The leaves (100 mg) were ground in 1 mL of phosphate buffer pH 7.5 and centrifuged at 15,000 *g* for 10 min at 4 ^ο^C. The supernatant was collected and analyzed for superoxide radical (O_2_ˉ) production (30 min at 37 °C), using xanthine as substrate, according to the kit instructions from the Amplex® Red Xanthine Oxidase Assay kit (Molecular Probes, Eugene, OR product number A22182). One enzyme unit was the amount of xanthine oxidase that will form 1 μmol of uric acid from hypoxanthine at 25 °C g^− 1^ on a leaf fresh weight basis [52].

### Hydrogen peroxide content

The leaves (100 mg) were ground in 1 mL of cold acetone and centrifuged at 5000 *g* for 10 min at 4 ^ο^C, and the supernatant was used for the H_2_O_2_ assay. The H_2_O_2_ content was measured using a one-step assay (Amplex® Red hydrogen peroxide/peroxidase assay kit; Invitrogen Molecular Probes, Inc., Eugene, OR, USA, product number A22188), which uses the Amplex® Red reagent (10-acetyl-3,7-dihydroxyphenoxazine), in combination with horseradish peroxidase (HRP), to detect H_2_O_2_. In the presence of peroxidase, the Amplex® Red reagent reacts with H_2_O_2_ in a 1:1 stoichiometry to produce the red-fluorescent oxidation product, resorufin, which has an absorption maximum at 560 nm. The background absorbance derived from a no-H_2_O_2_ control was subtracted for all samples and expressed as nmol g^− 1^ on fresh weight basis [53].

### Malondialdehyde content

Malondialdehyde content in leaf samples was measured using an OxiSelect thiobarbituric acid reactive substances (TBARS) assay kit (Cell Biolabs, San Diego, CA, USA, product number STA 330) as an estimate of lipid peroxidation. The TBARS assay is based on the reactivity of MDA with two molecules of thiobarbituric acid (TBA) via an acid-catalyzed nucleophilic-addition reaction. The resulting pinkish-red fluorescent MDA:TBA (1:2) adduct has an absorbance maximum at 532 nm and can be measured colorimetrically [54]. In the present study, the estimation of MDA content was done as explained by Narayanan et al. [25].

### Cell membrane damage

Cell membrane damage was measured according to Sairam et al. [55]. Leaf punches (~100 mg) were placed in 20 mL of deionized water in two test tubes. One tube was incubated in a water bath at a constant temperature of 40 ^ο^C for 30 min, and its conductivity (C_1_) was measured with a conductivity meter. The second tube was placed in a boiling water bath (100 ^ο^C) for 10 min, cooled, and conductivity was recorded (C_2_). Cell membrane damage (i.e., ion leakage) was expressed as a percentage using the formula: [1-(C_1_/C_2_)] × 100.

### Antioxidant enzyme activities

For SOD, CAT, POX, and glutathione peroxidase (GPX) enzyme assays, frozen leaf samples were homogenized in 1 mL of ice-cold 0.1 M Tris-HCl buffer, pH 7.8, per gram fresh weight. The homogenate was centrifuged at 20,000 g for 15 min at 4 ^ο^C. The supernatant was used for measuring enzyme activity.

### Superoxide dismutase enzyme activity

Total SOD activity was measured on the supernatant with a superoxide dismutase assay kit (Cayman Chemical, Ann Arbor, Michigan, USA, product number 706002) according to manufacturer’s instructions. This kit utilizes a tetrazolium salt for detection of superoxide radicals generated from oxygen by xanthine oxidase acting on hypoxanthine. One unit of SOD is the amount of enzyme needed to obtain 50% dismutation of superoxide radical on leaf fresh weight basis [56].

### Catalase enzyme activity

The catalase enzyme activity was measured using Amplex® Red catalase assay kit (Molecular Probes, Invitrogen, Inc., Eugene, OR, USA, product number A22180), as it provides an ultrasensitive, simple assay method for measuring CAT activity. In the assay, CAT first reacts with H_2_O_2_ to produce water and oxygen (O_2_). Next, the Amplex red reagent (10-acetyl-3,7-dihydroxyphenoxazine) reacts with a 1:1 stoichiometry with any unreacted H_2_O_2_ in the presence of HRP to generate the red-fluorescent oxidation product, resorufin, which has an absorption maximum at 560 nm. One enzyme unit was the amount of catalase enzyme that decomposes 1.0 μmol of H_2_O_2_ min^− 1^ g^− 1^ of tissue on leaf fresh weight basis at 25 °C [57].

### Peroxidase enzyme activity

The peroxidase (POX) enzyme activity was measured using a Amplex® Red hydrogen peroxide/peroxidase assay kit (Molecular Probes, Invitrogen, Inc., Eugene, OR, USA, product number A 22188), which is a one-step assay that uses the Amplex® Red reagent (10-acetyl-3,7-dihydroxyphenoxazine) to detect POX activity. The POX enzymatic activity was determined following the same procedure as the determination of H_2_O_2_ except that the Amplex Red reagent contained 2 mmol H_2_O_2_ instead of HRP. One enzyme unit is the amount of enzyme that will form 1.0 mg purpurogallin from pyrogallol in 20 s at pH 6.0 and 20 °C on leaf fresh weight basis [58].

### Glutathione peroxidase enzyme activity

The GPX was assayed using a Cayman’s GPX assay kit (Cayman Chemical, Ann Arbor, MI, product number 703102). The GPX activity was measured indirectly by a coupled reaction with glutathione reductase (GR). Oxidized glutathione (GSSG), produced upon reduction of hydroperoxide by GPX, is cycled to its reduced state by GR and NADPH. The oxidation of NADPH to NADP^+^ is accompanied by a decrease in absorbance at 340 nm. Under conditions in which the GPX activity is rate limiting, the rate of decrease in the absorbance at 340 nm is directly proportional to the GPX activity in the sample. To each well of a microplate, 100 μL of assay buffer (50 mM Tris-HCl, pH 7.6 containing 5 mM EDTA), 50 μL of co-substrate mixture (used as provided by manufacture, lyophilized powder of NADPH, glutathione and glutathione reductase) and 20 μL of sample was added. For background or non-enzymatic controls, 120 μL of assay buffer and 50 μL of co-substrate were added. Similarly, for positive controls, 100 μL of assay buffer, 50 μL of co-substrate mixture and 20 μL of diluted GPX (1:50; control) were added. The reaction was initiated by adding 20 μL of cumene hydroperoxide (used as supplied) to all wells. The microplate was carefully shaken for a few sec to mix the samples and reagent. The absorbance at 340 nm was read every min for 5 min using an Epoch spectrophotometer (BioTek, Winooski, VT, USA). The change in absorbance at 340 nm min^− 1^ was calculated for each sample. The absorbance by background was subtracted from the sample to get the actual change in absorbance due to the enzyme in the sample. One unit enzyme will oxidize 1 nmol of NADPH to NADP^+^ min^− 1^ on leaf fresh weight basis [59].

### Lipid extraction and ESI-MS/MS lipid profiling in leaves

At each temperature regime on 10th d of HT stress, the flag leaf was collected between 10:00 and 11:00 h. At sampling, the middle one third of each flag leaf was cut and immediately chopped into pieces and transferred to 6 mL of isopropanol with 0.01% BHT at 75 °C in a 50-mL glass tube with a Teflon-lined screw cap (Thermo Fisher Scientific, Inc., Waltham, MA, USA). Lipid extraction was performed as described by Narayanan et al. [25]. An automated electrospray ionization-tandem mass spectrometry approach was used for analysis. Polar and oxidized lipid profiling were carried out as described previously [25, 60]. Double bond index was calculated as per Narayanan et al. [25]. A response factor was applied to the galactolipids; data calculated in comparison to the saturated internal standards was divided by 2.8 to account for the greater response of the mass spectrometer to unsaturated lipids when analyzed as [M + NH_4_]^+^ adducts. Triacylglycerols were determined using the approach described by Li et al. [61]; response factor values were taken from Li et al. [61].

### Leaf anatomy using scanning electron microscope (SEM)

At each temperature regime on 10th d of HT stress, the flag leaf was harvested between 10:00 and 11:00 h and imaged soon after picking. The middle portion of flag leaf was cut into small pieces. The upper or lower portion of the leaf was mounted on double stick carbon tape affixed on a carbon stub and viewed with SEM. Similarly, the cross-section area of the leaf was mounted by affixing in vertical position and viewed with SEM (Nova NanoSEM 430, FEI, Hillsboro, OR) using a vCD detector (low voltage high Contrast Detector). The SEM was operated in a beam deceleration mode at a landing energy of 760 eV, under high vacuum, 3.5 kV, with a spot size of 2.5. The images were taken at 500, 1000, 2000 and 4000 x magnification. However, for viewing the cross section of the leaf, the SEM was operated under low vacuum mode, 3.5 kV, with a spot size of 4.0, and the image was captured using a vCD detector. Images depicting 10, 20, 30, 40 and 50 μm were obtained.

### Leaf anatomy using transmission electron microscope (TEM)

At each temperature regime on the 10th d of HT stress, a flag leaf was collected between 10:00 and 11:00 h. The leaf sample was fixed immediately in 4% glutaraldehyde in 100 mM sodium cacodylate buffer, pH 7.2. Following aldehyde fixation, samples were post-fixed in 2% osmium tetroxide in the same cacodylate buffer, stained in 1% aqueous uranyl acetate for 8 h, dehydrated in acetone, and embedded in Spurr’s resin. Ultrathin sections (< 100 nm thick) were made using an ultramicrotome, examined with a CM 100 TEM (FEI Company, Hillsboro, OR, USA), and photographed for image analysis with an AMT digital image capture system.

### Yield and its components

For each temperature treatment, the spikes of the main tiller of plants were tagged and used for calculating seed set percentage [4]. At maturity, plants were hand-harvested by cutting at the potting soil level. Vegetative parts and spikes (main spike and other spikes separately) were dried at 65 °C for 7 d and 40 °C for 10 d, respectively, for determination of dry weight. Grain number per main spike was counted manually. Seed set was determined as the ratio of florets with grain to the total number of florets, and expressed as a percentage [4]. The other spikes were also hand threshed to separate grains and counted manually. Seed size was calculated by dividing the total grain weight by number of grains.

### Statistical analyses

Data were analyzed using PROC GLM in the SAS software [62]. The experimental design was a randomized complete block. Plants for different treatments were selected randomly and randomly arranged within each growth chamber (treatments). Growth chamber temperatures were also randomly assigned. The data of each experiment were analyzed separately and in combination (pooled analysis) for physiological, biochemical and lipid traits. The results of both experiments separately or in combination had similar responses and significance for all traits, so data were combined for reporting. For all measured observations standard errors were shown as an estimate of variability, and means were separated using LSD at probability level of 0.05. For lipid analysis, significance was determined at *P* ≤ 0.05, after correcting for the false discovery rate, using Excel 2010. Comparisons were between stress samples and their controls. The lipids and grain yield data from OT and HT plants were uploaded to MetaboAnalyst (metabolanalyst.ca) for correlation analysis using Spearman’s rank method, to identify the lipid species that had the strongest relationship with grain yield. The top five lipid species having positive or negative relationships with grain yield were presented. The lipid species having highest positive or negative relationship were regressed against grain yield using SigmaPlot, version 14.0 and presented.

## Results

### Quality control of growth chambers

Mean daytime and nighttime temperatures in all growth chambers were ± 0.5 ^ο^C of the target day and night temperatures, and relative humidity was within ±10% of the target. Our earlier study showed that there is no variation in growth (plant height: 64.0 ± 0.9 cm; tiller numbers plant^− 1^: 3.5 ± 0.1; spike number per plant: 2.6 ± 0.1; and shoot biomass: 3.7 ± 0.2 g plant^− 1^) of the spring wheat genotype Pavon grown in these growth chambers set at 20/15 °C, 85% RH and 12 h photoperiod [63]. This implies that the growth chamber used for the present experiment had uniform environmental conditions. Apart from this, the same growth chambers were constantly checked for temperature by digital thermometer. The temperature control and growth chamber performance were previously described [63, 64].

### Effect of HT on physiological traits

**Fig. 1:**
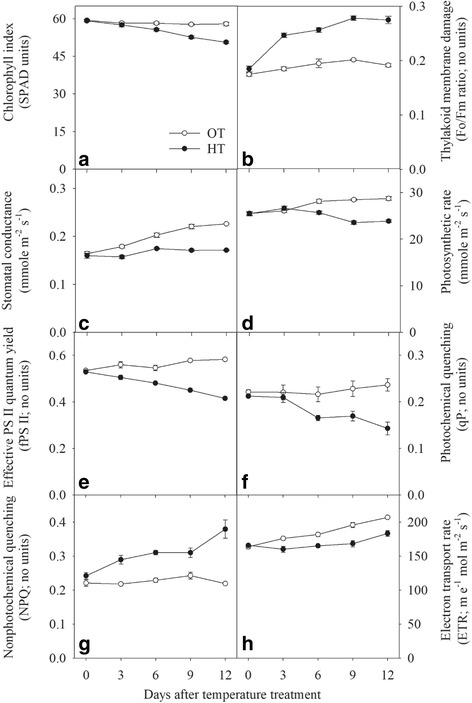
Effects of temperature (OT: 25/15 °C: daytime maximum/nighttime minimum temperature and HT: 35/25 °C) during booting stage of wheat genotype Chinese Spring on **a** chlorophyll index (SAPD units), **b** thylakoid membrane damage (Fo/Fm ratio; dimensionless), **c** stomatal conductance (mmol m^− 2^ s^− 1^), **d** photosynthetic rate (μmol m^− 2^ s^− 1^), **e** effective PSII quantum yield (dimensionless), **f** photochemical quenching (dimensionless), **g** non-photochemical quenching (dimensionless), and **h** electron transport rate (μmol e^− 1^ m^− 2^ s^− 1^) of wheat genotype Chinese Spring. Values shown are mean ± SE; *n* = 10 (two experiments with five replications each, combined). Vertical bars denote ± S.E. of means. OT, optimum temperature; HT, high temperature

Significant effects of temperature (*P* ≤ 0.001), day of observation (P ≤ 0.001) and interaction of temperature and day of observation (P ≤ 0.001) were observed for chlorophyll index (SPAD units), thylakoid membrane damage (Fo/Fm ratio), stomatal conductance (mmol m^− 2^ s^− 1^), photosynthetic rate (μmol m^− 2^ s^− 1^), effective PSII quantum yield (ϕ), photochemical quenching (qP) and non-photochemical quenching (NPQ), and electron transport rate (ETR; μ e^− 1^ mol m^− 2^ s^− 1^) (Fig. 1). The chlorophyll index and photosynthetic rate were lower, starting at 6 d of HT stress (Fig. 1a, d), whereas stomatal conductance was lower from 3 d of HT stress (Fig. 1c). The thylakoid membrane damage at HT was higher starting at 3 d of stress (Fig. 1b). Irrespective of day of observation, HT stress lowered the chlorophyll index, stomatal conductance and photosynthetic rate by 8, 8, and 16%, respectively (Fig. 1a–d). However, the thylakoid membrane damage was higher by 31% over OT (Fig. 1b). Regardless of days of observation, HT decreased the effective quantum yield (15%, Fig. 1e), photochemical quenching (20%, Fig. 1f), and electron transport rate (20%, Fig. 1h) in comparison with OT. In contrast, the non-photochemical quenching was higher at HT (Fig. 1g, 35%) compared with OT.

### Effect of HT on oxidant production, MDA content, membrane damage, and antioxidant enzyme activities

**Fig. 2:**
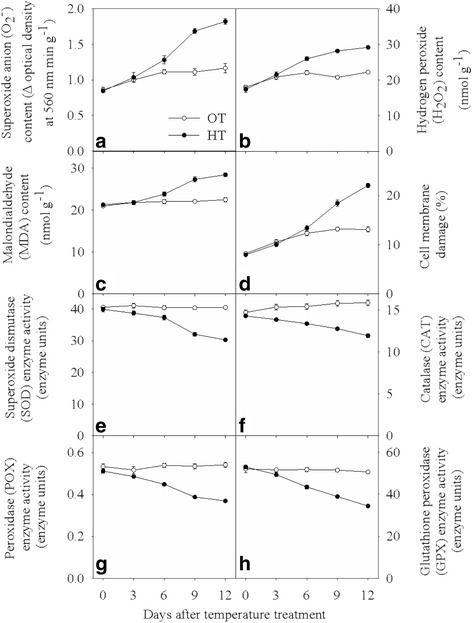
Effects of temperature (OT: 25/15 °C: daytime maximum/nighttime minimum temperature and HT: 35/25 °C) during booting stage on **a** superoxide radical content (Δ optical density min^− 1^ g^− 1^), **b** hydrogen peroxide content (nmol g^− 1^), **c** malondialdehyde content (nmol g^− 1^), **d** cell membrane damage (%), **e** superoxide dismutase enzyme activity (enzyme units), **f** catalase enzyme activity (enzyme units), **g** peroxidase enzyme activity (enzyme units) and **h** glutathione peroxidase enzyme activity (enzyme units) of wheat genotype Chinese Spring. Values shown are mean ± SE; *n* = 10 (two experiments and five replications). Vertical bars denote ± S.E. of means. OT, optimum temperature; HT, high temperature

Significant differences in O_2_ˉ, H_2_O_2_, and MDA concentrations were observed beginning at 6 d of HT stress (Fig. 2a–c). Significant differences in cell membrane damage were observed beginning at 9 d of HT stress (Fig. 2d). O_2_ˉ content (*P* ≤ 0.001), H_2_O_2_ content (P ≤ 0.001), MDA formation (P ≤ 0.001), and cell membrane damage (P ≤ 0.001) were higher at HT than OT (Fig. 2a, b, c, and d). Averaged across all dates of observation, HT stress increased O_2_ˉ and H_2_O_2_ content by 27 and 18%, respectively, compared with OT (Fig. 2a, b). Likewise, HT increased MDA and cell membrane damage by 12 and 25%, respectively, compared with OT (Fig. 2c, d). Significant (P ≤ 0.001) differences in SOD, CAT, POX, and GPX enzyme activities were observed from 3 d of HT stress. Overall, HT stress significantly decreased SOD, CAT, POX and GPX enzyme activities by 12, 14, 17, and 15%, respectively, compared with OT (Fig. 2e–h).

### Effect of HT on polar lipids and TAG

**Fig. 3:**
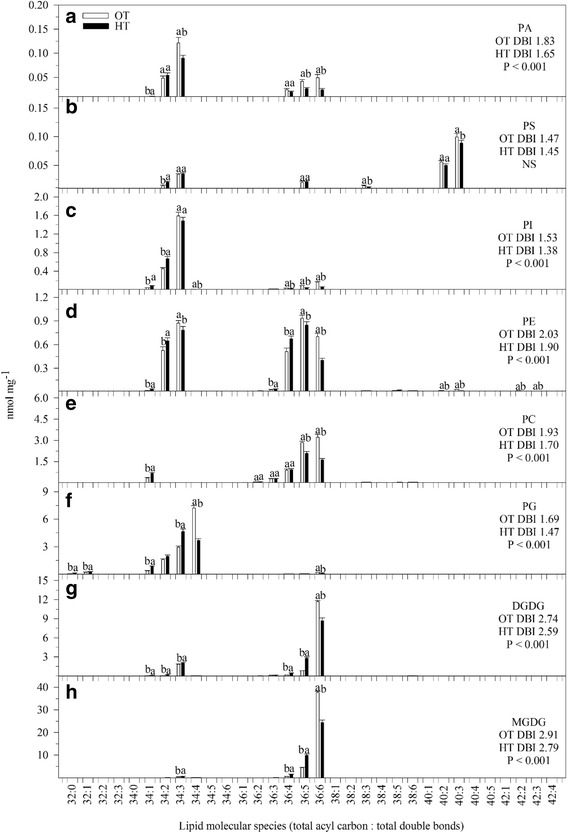
Effects of temperature (OT: 25/15 °C: daytime maximum/nighttime minimum temperature and HT: 35/25 °C) during booting stage on polar lipid molecular species of wheat genotype Chinese Spring (**a**) PA, phosphatidic acid, (**b**) PS, phosphatidylserine; (**c**) PI, phosphatidylinositol; (**d**) PE, phosphatidylethanolamine; (**e**) PC, phosphatidylcholine; (**f**) PG, phosphatidylglycerol; (**g**) DGDG, digalactosyldiacylglycerol and (**h**) MGDG, monogalactosyldiacylglycerol. Sampling was on day 10 of the treatment. Values shown are mean ± SE; *n* = 10 (two experiments with five replications each, combined). Vertical bars denote ± S.E. of means. Means with different letters are significantly different according to the least significant difference (LSD) test at *P* < 0.05. OT, optimum temperature; HT, high temperature; DBI, double bond (unsaturation) index

**Fig. 4:**
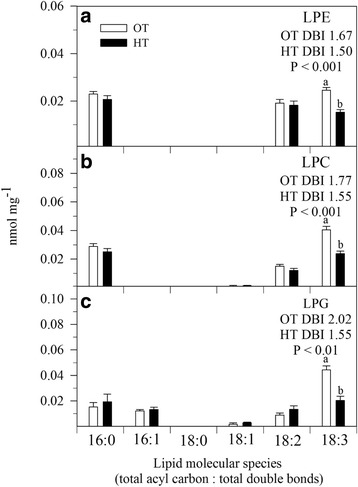
Effects of temperature (OT: 25/15 °C: daytime maximum/nighttime minimum temperature and HT: 35/25 °C) during booting stage on lysolipid molecular species of wheat genotype Chinese Spring. (**a**) LPE, lysophosphatidylethanolamine; (**b**) LPC, lysophosphatidylcholine; and (**c**) LPG, lysophosphatidylglycerol. Sampling was on day 10 of the treatment. Values shown are mean ± SE; *n* = 10 (two experiments and five replications each, combined). Vertical bars denote ± S.E. of means. Means with different letters are significantly different according to the least significant difference (LSD) test at *P* < 0.05. OT, optimum temperature; HT, high temperature; LPG, lysophosphatidylglycerol; DBI, double bond (unsaturation) index

**Fig. 5:**
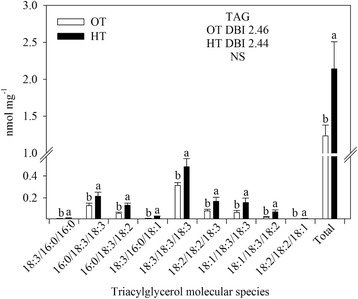
Effects of temperature (OT: 25/15 °C: daytime maximum/nighttime minimum temperature and HT: 35/25 °C) during booting stage on triacylglycerol (TAG) in leaves of wheat genotype Chinese Spring. Sampling was on day 10 of the treatment. No positional specificity of the fatty acyl chains on the glycerol was determined, nor is implied. Values shown are mean ± SE; *n* = 10 (two experiments and five replications each, combined). Vertical bars denote ± S.E. of means. Means with different letters are significantly different according to the least significant difference (LSD) test at *P* < 0.05. OT, optimum temperature; HT, high temperature

HT stress resulted in significant decreases in monogalactosyldiacylglycerol (MGDG) and phosphatidylglycerol (PG), phosphatidylcholine (PC) and phosphatidic acid (PA). The lysophospholipids, lysophosphatidylglycerol (LPG), lysophosphatidylcholine (LPC), and lysophosphatidylethanolamine (LPE), significantly (*P* ≤ 0.05) decreased under HT stress compared with OT (Table 1). HT stress caused a decrease in the amount of more unsaturated lipid species and an increase in the amount of less unsaturated lipid species (Fig. 3). Many species containing two polyunsaturated acyl chains, such as 36:6 (di18:3) species of MGDG, DGDG, PG, PC, PE, PI and PA, 36:5 (18:3/18:2) species of PC, PE, PI, and PA, and 34:4 (18:3/16:1) species of PG and PI decreased under HT stress (Fig. 3). On the other hand, the amount of less unsaturated acyl chains, including 36:5 (18:3/18:2 combination) and 36:4 (18:2/18:2 or 18:3/18:1 combination) species of MGDG and DGDG, 34:3 (18:3/16:0 or 18:2/16:1) species of PG and PA, 34:2 (18:2/16:0) species of PE, PI, and PS, and 34:1 (18:1/16:0 or 18:0/16:1) species of DGDG, PG, PC, PE, PI, and PA, increased under HT stress (Fig. 3). These changes led to a decrease in the unsaturation index of most polar lipid classes at HT stress (Fig. 3a, c, d, e, f, g, and h). HT stress decreased the 18:3 acyl species of lysoPC, lysoPE, and lysoPG compared with OT (Fig. 4). A significant (*P* ≤ 0.05) increase in total TAG content was observed under HT stress compared with OT, without a change in unsaturation index (Fig. 5). Acylated MGDG (acMGDG) species are formed by acylation of MGDG on the carbon at the 6-position of galactose (Additional file 1: Figure S1). Acylated MGDG amounts were increased by high temperature stress (Additional file 1: Figure S1).

**Table 1:** Total amount of lipid in each head group class in wheat leaves under OT optimal temperature and HT high temperature. Values are means ± standard error (SE; *n* = 10)

Polar lipid	OT	HT
	Lipid per dry weight (nmol mg^− 1^)	
MGDG	43.3^a^ ± 2.0	36.5^b^ ± 5.4
DGDG	14.8^a^ ± 0.17	14.4^a^ ± 0.86
PG	12.7^a^ ± 0.5	11.7^b^ ± 0.7
PC	15.3^a^ ± 0.009	13.5^b^ ± 0.89
PE	3.7^a^ ± 0.2	3.5^a^ ± 0.20
PI	2.4^a^ ± 0.1	2.4^a^ ± 0.1
PA	0.29^a^ ± 0.03	0.23^b^ ± 0.02
PS	0.28^a^ ± 0.01	0.27^a^ ± 0.01
LPG	0.082^a^ ± 0.1	0.069^b^ ± 0.01
LPC	0.085^a^ ± 0.006	0.062^b^ ± 0.005
LPE	0.067^a^ ± 0.004	0.054^b^ ± 0.004
Total polar lipid	93.2^a^ ± 4.2	83.0^b^ ± 9.2

*MGDG* monogalactosyldiacylglycerol, *DGDG* digalactosyldiacylgylcerol, *PG* phosphatidylglycerol, *PC* phosphatidylcholine, *PE* phosphatidylethanolamine, *PI* phosphatidylinositol, *PA* phosphatidic acid, *PS* phosphatidylserine, *LPG* lysophosphatidylglycerol, *LPC* lysophosphatidylcholine, *LPE* lysophosphatidylethanolamine. All polar lipid classes were represented as mean ± SEM, and the same lower case letter indicates no significant difference at LSD (α < 0.05) level between *OT* optimal temperature, and *HT* high temperature; different letters indicate a significant difference

### Effect of HT on molecular species with oxidized fatty acyl chains (oxidized lipids)

**Fig. 6:**
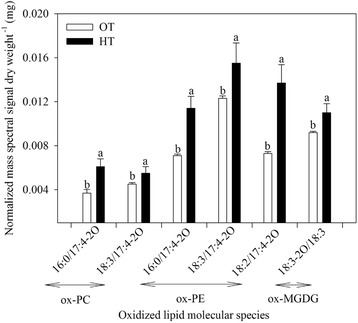
Effects of temperature (OT: 25/15 °C: daytime maximum/nighttime minimum temperature and HT: 35/25 °C) during booting stage on content of lipids containing oxidized fatty acids (oxidized lipids) in wheat genotype Chinese Spring. Sampling was on day 10 of the treatment. No positional specificity of the fatty acyl chains is implied. Values shown are mean ± SE; *n* = 10 (two experiments and five replications each, combined). Vertical bars denote ± S.E. of means. Means with different letters are significantly different according to the least significant difference (LSD) test at *P* < 0.05. OT, optimum temperature; HT, high temperature; PC, phosphatidylcholine; PE, phosphatidylethanolamine; MGDG, monogalactosyldiacylglycerol

HT stress caused significant (P ≤ 0.05) variation in putative membrane lipids putatively containing oxidized acyl chains (Fig. 6). Species of oxidized PC, PE, and MGDG were observed. Oxidized chain identification by mass spectrometry was based on detection of oxidized lipids’ nominal masses, and in some cases, water losses detected by mass spectrometry of the fatty acyl chains [25]. The acyl species are indicated by “acyl carbons:double bond equivalents beyond the acid carbonyl-number of oxygens in addition to the carbonyl group”. For example, 18:3-2O, indicates three double bond equivalents and two oxygens beyond the carbonyl group; 18:3-2O might be a hydroperoxide or a ketol derived from 18:3. Similarly, 17:4-2O indicates a 17-carbon fatty acid with four double bond equivalents and two oxygens in addition to those in the carbonyl group. Its chemical formula was defined previously by accurate mass analysis of wheat leaf extracts [25], but the structure of this modified fatty acid is not known. The galactolipid species 18:3-2O/18:3 MGDG was increased significantly (*P* < 0.05) under HT stress (Fig. 6). The 16:0/17:4-2O and 18:3/17:4-2O ox-PC lipid species were increased by ~1.6 and 1.2-fold, respectively by HT stress (Fig. 6). Similarly, the 16:0/17:4-2O and 18:2/17:4-2O-containing PE were increased ~1.6 and 1.9-fold, respectively under HT stress (Fig. 6).

### Effect of HT on leaf anatomy

**Fig. 7:**
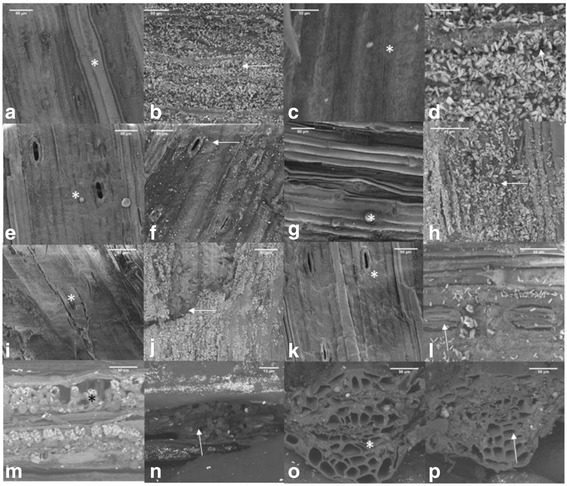
Effects of temperature (OT: 25/15 °C: daytime maximum/nighttime minimum temperature and HT: 35/25 °C) during booting stage on leaf surface morphology. Abaxial surface of OT leaf (**a**, **c**, **e**) and HT stressed leaf (**b**, **d**, **f**) showing the disintegration of wax and closure of stomata. Similarly, the adaxial surface of OT (**g**, **i**, **k**) and HT stressed leaf (**h**, **j**, **l**) showing the integration of wax and closure of stomata. Arrows indicate disintegrated wax and closed stomata under HT stress. The corresponding information in OT is indicated by *. The decreased mesophyll thickness under HT stress is shown in (**n**) and its corresponding OT was shown as (**m**). The vascular bundle size and morphology of OT and HT was shown in (**o**) and (**p**), respectively. OT, optimum temperature; HT, high temperature

HT stress caused disintegration of the epicuticular wax layer and formed small rod-like structures on both the abaxial (Fig. 7b, d) and adaxial (Fig. 7h, j) surfaces of leaves, while at OT the epicuticular wax was intact (Fig. 7a, e, g, i). SEM showed no difference in the wax morphology between the abaxial (Fig. 7b, d) and the adaxial (Fig. 7h, j) sides of the HT stressed leaf. Under HT stress, the adaxial epicuticular and cuticular wax layers had apparently fragmented, exposing more mesophyll tissue (Fig. 7j), compared with OT (Fig. 7i). HT stress caused complete to partial closure of stomata (Fig. 7f), while at OT the stomata were fully open (Fig. 7e). HT stress decreased the leaf thickness (Fig. 7n), number of phloem cells and tracheids, and increased the suberization of vessels (Fig. 7p) compared with OT (Fig. 7m, o).

**Fig. 8:**
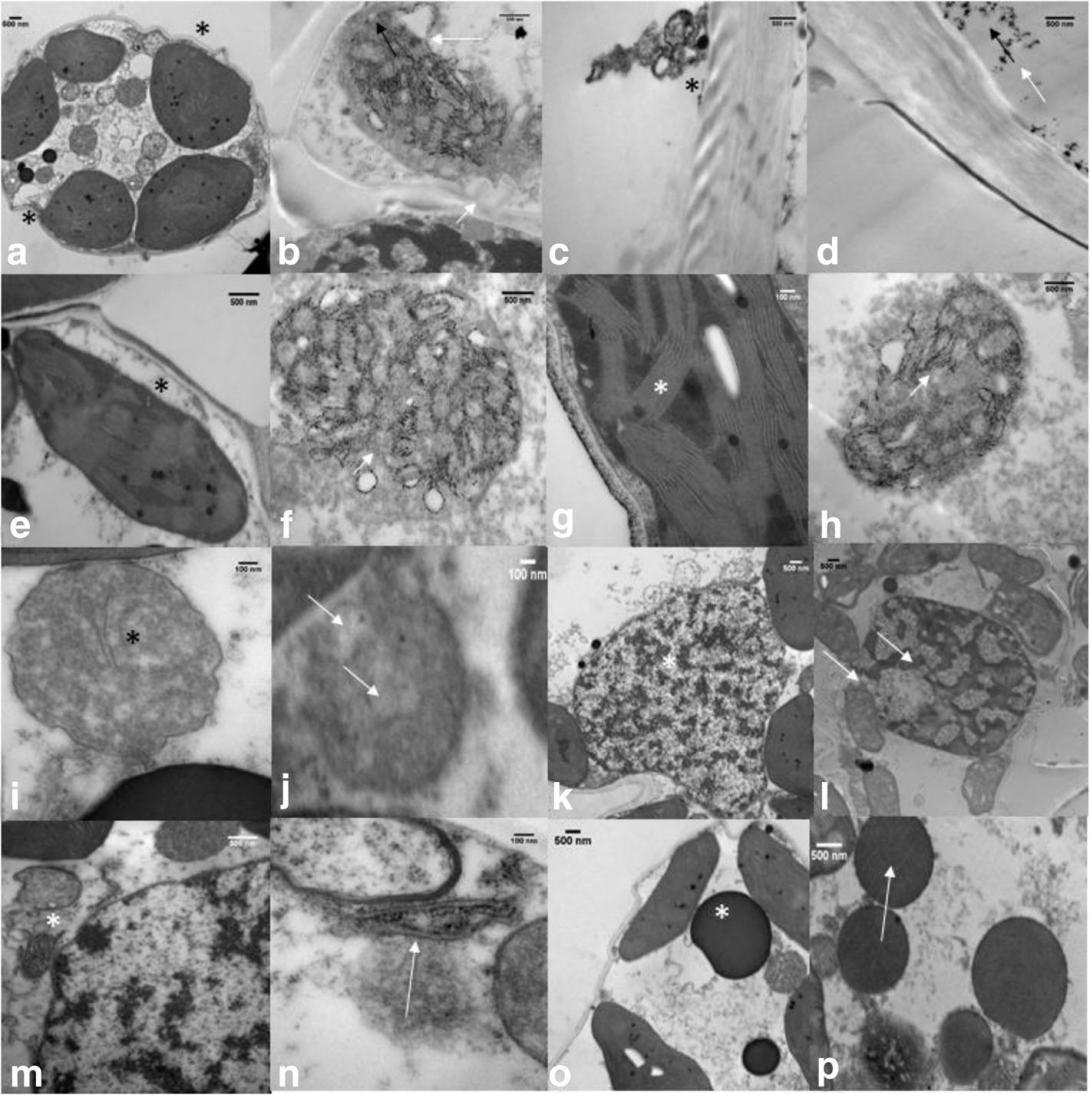
Effects of temperature (OT: 25/15 °C: daytime maximum/nighttime minimum temperature and HT: 35/25 °C) during booting stage on leaf ultrastructure. The OT (**a**, **c**, **e**, **g**, **i**, **k**, **m**, **o**) and HT (**b**, **d**, **f**, **h**, **j**, **l**, **n**, **p**) leaf images were showing the normal (*) and abnormal or damaged (arrow) cell organelles. The image (**b**) showing the disintegration of cellular membrane, (**d**) disintegration of wax layer, (**f**) disintegration of chloroplast membrane, (**h**) disintegration of thylakoid membrane, (**j**) disintegration of mitochondrial membrane. The image (**l**) showing the heterochromation, (**n**) detached endoplasmic reticulum, and (**p**) plastoglobule accumulation under HT stress. The corresponding normal structure is shown as *. OT, optimum temperature; HT, high temperature

Analysis of the cellular ultrastructure using TEM indicates that HT stress caused damage to the plasma membrane (Fig. 8b) and the epicuticular layer (Fig. 8d) compared with OT (Fig. 8a, c). The chloroplast and thylakoid membranes were damaged under HT stress (Fig. 8f, h) compared with OT (Fig. 8e, g). Similarly, the mitochondrial membrane and cristae were damaged under HT stress (Fig. 8j), as compared with OT (Fig. 8i). Damaged and discontinuous nuclear envelope and heterochromatin were observed under HT stress (Fig. 8l), whereas, in OT, the nuclear membrane was continuous and euchromatin was observed (Fig. 8k). The rough endoplasmic reticulum (ER) was attached to the nuclear envelope and is coiled under OT (Fig. 8m), while, in HT stress, rough ER was not attached to the nuclear envelope and appeared to be linear (Fig. 8n). The number and size of plastoglobules were greater in HT stress (Fig. 8p) compared with OT (Fig. 8o).

### Effect of HT on seed yield and its components

**Fig. 9:**
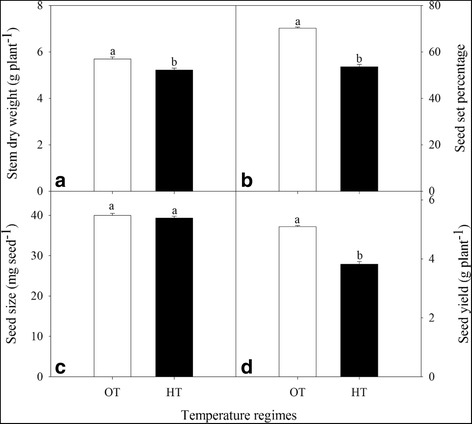
Effects of temperature (OT: 25/15 °C: daytime maximum/nighttime minimum temperature and HT: 35/25 °C) during booting stage on (**a**) stem dry weight (g plant^− 1^), (**b**) seed-set percentage, (**c**) seed size (mg seed^− 1^), and (**d**) seed yield (g plant^− 1^) of wheat genotype Chinese Spring. Values shown are mean ± SE; *n* = 20 (two experiments and ten replications each, combined). Vertical bars denote ± S.E. of means. Means with different letters are significantly different according to the least significant difference (LSD) test at *P* < 0.05. OT, optimum temperature; HT, high temperature

HT stress resulted in a significant (*P* ≤ 0.05) decreases in stem dry weight and yield components in the primary spike compared to OT (Fig. 9). The seed set and seed yield per plant were decreased by ~24% (Fig. 9b, d) and stem dry weight (Fig. 9a) was decreased by 8%.

### Lipids, HT, and yield

A Spearman’s correlation analysis among yield and lipid levels across the OT and HT samples indicated that several lipids were associated with high yield (Table 2). These lipids were also highest at OT. For example, PC(36:6), with two triply unsaturated fatty acids, was a marker positively associated with optimal conditions and high yield (Fig. 3e), while the not-fully-polyunsaturated MGDG(36:4) was associated with HT and low yield (Fig. 3h). The relationship of levels of these lipids to yield for individual HT and OT samples is shown in Additional file 2: Figure S2a, b.

**Table 2:** Top five lipid species correlated with grain yield plant^−1^ under OT optimum temperature and HT high temperature conditions

Relationship	Lipid species	Spearman’s correlation coefficient
Lipids positively associated with yield	PC(36:6)	0.85
	LysoPC(18:3)	0.84
	PE(42:3)	0.84
	PC(34:4)	0.81
	PI(36:6)	0.79
Lipids negatively associated with yield	MGDG(36:4)	−0.94
	MGDG(36:3)	−0.92
	PG(32:1)	−0.92
	MGDG(34:3)	−0.92
	DGDG(34:1)	−0.91

*OT* optimum temperature, *HT* high temperature

## Discussion

Taken together, the data indicate that HT stress caused decreased unsaturation of phospholipids and accumulation of TAGs. Increased levels of some oxidized lipids were observed along with increased ROS and lower levels of enzymes that remove ROS. Similarly, unsaturated lipids, particularly those containing trienoic fatty acids were decreased, providing both the potential to reduce lipid oxidation but also loss of a potential sink for ROS [15, 18]. Damaged cell membranes, organelles, and wax surfaces were observed, along with a decreased photosynthetic rate, which led to a low biomass and yield in plants subjected to HT.

Lipid changes undoubtedly represent a mixture of effects. Desaturase activity is reduced in response to HT and these changes are adaptive in terms of maintaining the fluidity of the membrane environment (Fig. 3) [35, 36, 65–71]. It’s likely that the accumulation of TAGs (Fig. 5), which may occur in observed plastoglobules (Fig. 8) and/or cytosolically, includes acyl chains removed from membrane lipids being remodeled under stress. The lack of difference in unsaturation index of TAGs between leaves of OT and HT plants is consistent with this interpretation.

Peroxidation of lipids in plants can occur by via lipoxygenases (LOXs), by singlet oxygen generated by photosynthesis, or by radicals [15, 72, 73]. In leaves in the light, most primary lipid peroxidation is by reaction with singlet oxygen, a high energy form of oxygen formed when an excited chlorophyll molecule transfers energy to ground state O_2_ [74]. In wheat, galactolipids (MGDG and DGDG) are the major chloroplast lipids, and MGDG (36:6) and DGDG (36:6) (each with two 18:3 chains) are the major molecular species. These “36:6” species are particularly vulnerable to peroxidation by singlet oxygen and by LOX. Indeed, MGDG (18:3-2O/18:3), which is consistent with being a peroxidized species, is increased in HT (Fig. 6). Peroxidized trienoic fatty acids can undergo chain fragmentation and the pool of peroxides can be amplified; one of the lipid fragmentation products is MDA [14, 15], which was observed to increase in HT. Indeed, the observed greyish to dark material in the thylakoid (Fig. 8b–h) suggests decomposition of thylakoid membrane lipids [75], and the decrease in ϕ PSII ratio suggests the occurrence of photoinhibition (Fig. 1e) [76], leading to increased ROS generation and oxidative damage [76–78]. The cumulative result of abiotic stress is generation of three ROS species, singlet oxygen, superoxide radical, and hydrogen peroxide [74, 79]. Interestingly, recent work by the Farmer and Mueller groups demonstrates that trienoic-containing MGDG may be the sink for MDA and well as its source [15, 18]. Thus, the ongoing lowering of “36:6” galactolipid species levels, as MDA is produced, could potentially contribute to increased MDA levels.

Limitations to CO_2_ assimilation cause an imbalance between photochemical activity at photosystems and the electron requirement for photosynthesis [78, 80–82]. Physical damage to thylakoid and chloroplast membranes was observed under HT stress (Fig. 8f, h). As observed here, thylakoid membranes have been demonstrated to be more sensitive to HT stress than the chloroplast envelope or other cell compartments [83]. Indeed, excessive ROS accumulation damages pigments and proteins, as well as lipids [84], thereby contributing to oxidative damage. Chlorophyll is primarily located in the thylakoid membranes, where it forms complexes with the proteins of PSII and PSI and damage to thylakoid membranes may lead to chlorophyll loss [78, 82, 85].

Increased ROS content observed under HT is also associated with decreased antioxidant enzyme activities (Fig. 2a-h). Under normal conditions, the rate of production of ROS from electron transport chains is kept under control [12] by antioxidants and antioxidant enzymes. However, if damage to the thylakoid membranes and/or to the cristae, occurs, then the rate of production of ROS is increased [12], leading to the steady increase in MDA, cell membrane damage, and oxidative damage (Fig. 2c). Under HT stress, the balance between ROS and antioxidant enzyme activity is perturbed (Fig. 2a-h). SOD is usually considered to be the first line of defense against oxidative stress [12, 13]. High SOD activity can efficiently remove O_2_ˉ, leading to the production of H_2_O_2_ and its scavenging by CAT, POX and GPX [12, 13]. HT stress decreased all the above antioxidant enzymes activity (Fig. 2e–h) compared with OT. The current results suggest that activity of the antioxidant enzymes in chloroplasts may be downregulated by HT stress, exacerbating accumulation of ROS.

An increase in growth temperature has been directly linked to a decrease in photosynthetic rate (Fig. 1d) [86, 87] by both stomatal and nonstomatal limitations [78, 88], as shown by closed stomata and decreased chlorophyll content, PSII activity, mesophyll cell density, and damaged chloroplast structure [81, 82, 85, 89, 90]. Excess energy that cannot be used to drive photosynthesis enhances the production of ROS and induces photooxidative damage [77, 91]. An increase in Fo (Fig. 1b) and decrease in ϕ PSII (Fig. 1e) indicates damaged PSII reaction centers [88, 92, 93] and impeded transfer of excitation energy from the antenna to the reaction centers [80, 94]. HT stress decreased qP (Fig. 1f) and ETR (Fig. 1h) by 20%, indicating both donor and acceptor sides of the reaction center were affected by HT stress [78, 80, 81]. The NPQ increased under HT stress, compared to OT, demonstrating absorbed light energy is not utilized for production of ATP and NADPH_2_; instead absorbed energy is dissipated as heat [93, 95]. Apart from this, increased thylakoid membranes damage by HT causes proton leakage, leading to decreased NADPH_2_ production [78]. All these data clearly show occurrence of photoinhibition during HT stress. If this is the case, generation of ROS during photoinhibition could cause chlorophyll degradation, damage to PSII components, and inactivation of enzymes involved in CO_2_ assimilation [96] and could further explain the reductions in photosynthetic rate in HT stressed plants in the present study.

HT stress decreased the seed set percentage, grain number per spike, and grain yield per spike (Fig. 9). The decrease in grain number was due to decreased seed set [10, 43, 97]. Our previous study in wheat indicates that HT stress caused structural abnormalities to both pollen and pistil [4]. Hence, the decreased seed set may be due to malfunction of both pollen and pistil. The individual grain weight was not influenced by HT stress, because the stress was imposed during booting to anthesis stage. Hence, the decreased grain yield per spike is due to decreased grain number per panicle and decreased photosynthetic rate leading to reduced biomass accumulation.

Chinese Spring was selected for the current study because of its sensitivity to HT stress [98]. It has been widely used in genomic research and its genome is sequenced [99]. There is relatively little information that would allow comparison of lipids and their relationships with HT stress among wheat genotypes, although one study suggests that lipid compositions do vary among genotypes that differ in HT tolerance, and it is reasonable to postulate that this is related to stress tolerance [25].

## Conclusions

The present study has shown that the decrease in photosynthetic rate under HT stress is an interplay between thylakoid membrane damage, thylakoid membrane lipid composition, oxidative damage of cell organelle, and stomatal and non-stomatal limitations. Under HT stress accumulation of TAGs without change in unsaturation index as plastoglobules indicates lipid remodeling under stress. The changes in lipid species indicates increases in activities of desaturating, oxidizing, glycosylating and acylating enzymes under HT stress. Cumulative effect of HT stress is generation of ROS, cell organelle damage, plasma and cell organelle membrane damage and reduced antioxidant enzyme activity indicating the imbalance between ROS and antioxidant defense system. The oxidized lipid species identified from this study can be used as a biochemical marker for development of HT stress tolerant wheat genotype. Currently, newer genomic related tools are being used to identify the key target genes that respond to HT stress to facilitate the selection of HT stress tolerant lines. However, comprehensive expression studies on genes involved in thylakoid lipid synthesis, degradation, remodeling will provide more insight into mechanism of tolerance and provide opportunities to direct the wheat breeding program for HT stress tolerance.

## Additional files

Additional file 1: **Figure S1**. Effect of high temperature stress on galactose acylation of MGDG species in wheat. Sampling was on day 10 of the treatment. No analysis was performed to indicate the specific positions of the individual acyl chains in the acylated MGDG, which has the usual glycerol-linked fatty acids, plus an additional chain esterified to the galactose [100]. (DOCX 263 kb)

Additional file 2: **Figure S2**. Relationship between (a) PC(36:6) lipid species levels with grain yield plant^− 1^ and (b) MGDG(36:4) lipid species levels with grain yield plant^− 1^. (DOCX 222 kb)

## Abbreviations

DBI: Double bond (unsaturation) index
DGDG: Digalactosyldiacylgylcerol
HT: High temperature
LPC: Lysophosphatidylcholine
LPE: Lysophosphatidylethanolamine
LPG: Lysophosphatidylglycerol
MGDG: Monogalactosyldiacylglycerol
OT: Optimum temperature
PA: Phosphatidic acid
PC: Phosphatidylcholine
PE: Phosphatidylethanolamine
PG: Phosphatidylglycerol
PI: Phosphatidylinositol
PS: Phosphatidylserine
TAG: Triacylglycerol
