# Supplementary material for: Decreased photosynthetic rate under high temperature in wheat is due to lipid desaturation, oxidation, acylation, and damage of organelles
Source: BMC Plant Biol. 2018 Apr 5;18:55. doi: 10.1186/s12870-018-1263-z (PMC5887265; doi:10.1186/s12870-018-1263-z)

**Additional file 1.** Effect of high temperature stress on galactose acylation of MGDG species in wheat. Sampling was on day 10 of the treatment. No analysis was performed to indicate the specific positions of the individual acyl chains in the acylated MGDG, which has the usual glycerol-linked fatty acids, plus an additional chain esterified to the galactose [93].


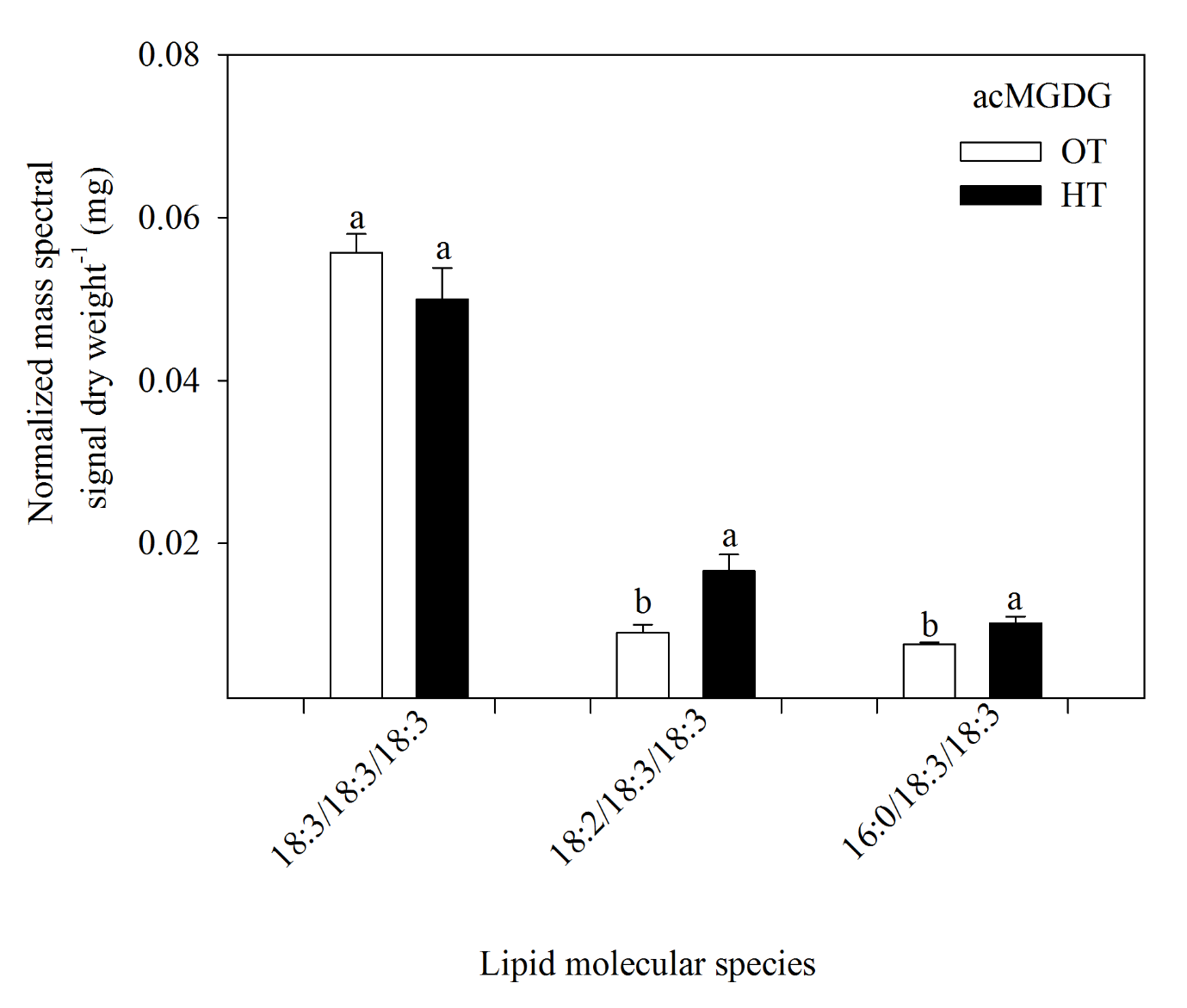

Supplement: Supplementary file 1 — Figure S1. Effect of high temperature stress on galactose acylation of MGDG species in wheat. Sampling was on day 10 of the treatment. No analysis was performed to indicate the specific positions of the individual acyl chains in the acylated MGDG, which has the usual glycerol-linked fatty acids, plus an additional chain esterified to the galactose [100]. (DOCX 263 kb) [file 12870_2018_1263_MOESM1_ESM.docx]
