# Supplementary material for: Decreased photosynthetic rate under high temperature in wheat is due to lipid desaturation, oxidation, acylation, and damage of organelles
Source: BMC Plant Biol. 2018 Apr 5;18:55. doi: 10.1186/s12870-018-1263-z (PMC5887265; doi:10.1186/s12870-018-1263-z)

**Additional file 2.** Relationship between (a) PC(36:6) lipid species levels with grain yield plant^-1^ and (b) MGDG(36:4) lipid species levels with grain yield plant^-1^. Filled circles represent HT samples and open circles represent OT samples.


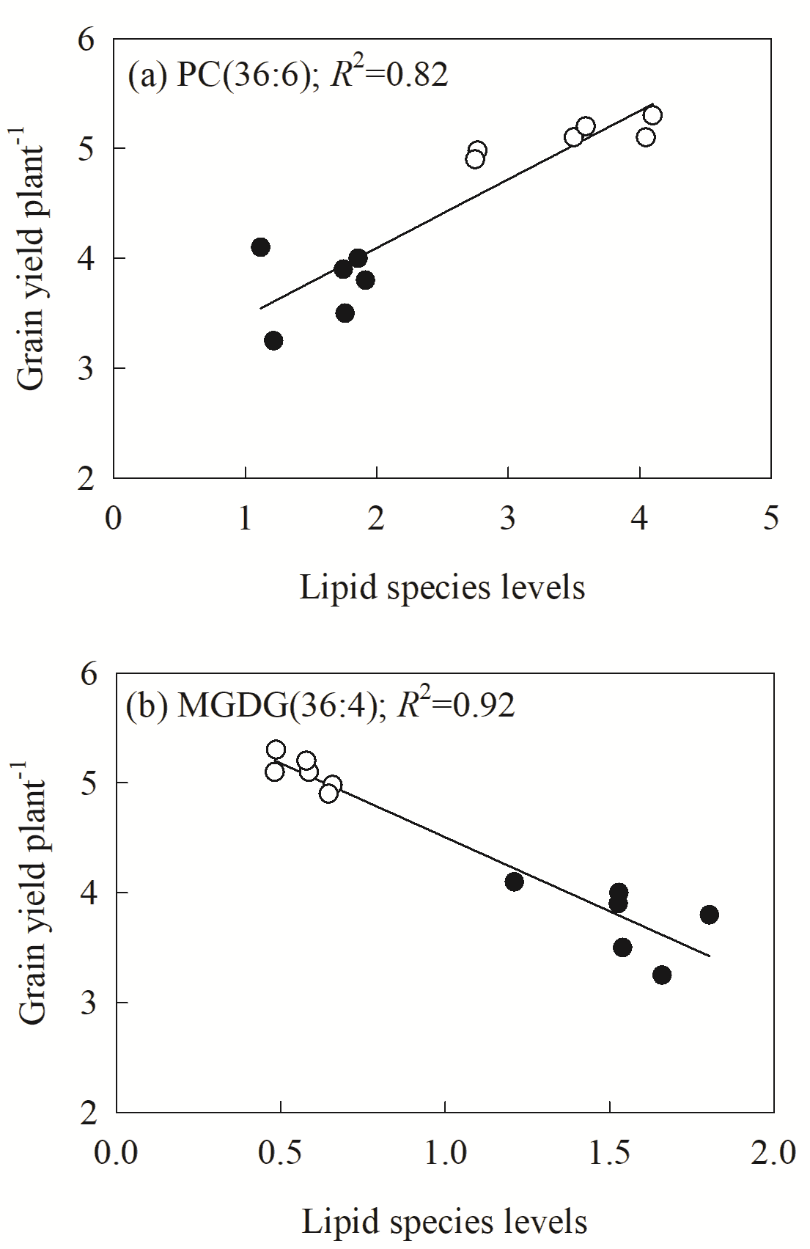

Supplement: Supplementary file 2 — Figure S2. Relationship between (a) PC(36:6) lipid species levels with grain yield plant− 1 and (b) MGDG(36:4) lipid species levels with grain yield plant− 1. (DOCX 222 kb) [file 12870_2018_1263_MOESM2_ESM.docx]
